# Factors related to negative feelings experienced by emergency department patients and accompanying persons: an Israeli study

**DOI:** 10.1186/s13584-017-0200-1

**Published:** 2018-01-04

**Authors:** Simha F. Landau, Judy Bendalak, Gila Amitay, Ohad Marcus

**Affiliations:** 1 Mildred and Benjamin Berger Professor Emeritus of Criminology at the Institute of Criminology, Faculty of Law, Hebrew University, Mt. Scopus, 91905 Jerusalem, Israel; 2 Department of Criminology, Western Galilee Academic College, Acre and Institute of Criminology, Faculty of Law, Hebrew University of Jerusalem, Jerusalem, Israel; 3 Department of Criminology, Max Stern Academic College of Emek Yezreel, Jezreel Valley Regional Council and Schools of Education and Social Work, Hebrew University of Jerusalem, Jerusalem, Israel; 4 Max Stern Academic College of Emek Yezreel, Jezreel Valley Regional Coumncil, Israel

**Keywords:** Patient satisfaction, Hospital violence, Emergency departments, Interpersonal skills, Waiting time

## Abstract

**Background:**

Studies on hospital violence have emphasized the importance of staff- service recipient interaction in leading to violent incidents. These incidents are the extreme result of service recipients’ frustration and anger in their interaction with staff.

The aim of this study was to analyze factors related to negative experiences of emergency department (ED) patients and accompanying persons in Israeli hospitals.

**Methods:**

Structured interviews with 692 participants in seven major general Israeli hospitals: 322 patients and 370 accompanying persons.

**Results:**

Negative feelings while in the ED were reported by 23.6% of patients and 20.5% of accompanying persons. Eight aggregate variables relating to staff-patients/accompanying persons interaction were identified: 1. General attitudes of staff and quality of ED experience; 2. Staff attitudes towards patients; 3. Staff attitudes towards accompanying persons; 4. Waiting; 5. Quality of perceived medical care; 6. Information provided to patients and accompanying persons; 7. Information provided to patients, as reported by accompanying persons; and 8. Severity of medical problem. Among patients, the only significant aggregate variable related to anger and frustration was perceived quality of care. Among accompanying persons, the three significant contributors to negative feelings were: 1. Staff’s general attitudes; 2. Attitudes towards patients; and 3. Severity of patients’ medical problem. Analysis of specific items within the variables revealed that, whereas patients’ negative feelings were related to nurses’ perceived negative attitudes those of accompanying persons were related to the doctors’ perceived negative attitudes. In addition, patients’ negative feelings were related to low severity of medical problem, whereas accompanying persons’ negative feelings were related to patients’ low severity of pain.

**Conclusions:**

The study reveals the importance of including both patients and accompanying persons in the analysis of staff-service recipient interactions in EDs. The results are discussed in terms of patients’ and accompanying persons’ different perspectives. Three practical implications of the results are put forward, aiming at reducing patients/accompanying persons-staff frictions in the EDs, thus decreasing the potential of violent outbursts against ED staff: (1) implementing a framework based on “patient-centeredness” for the restoration of patient’s sense of agency and empowerment; (2) broadening the scope of laws concerning patient’s rights to include their families and other accompanying persons; and (3) implementing courses on interpersonal and human service skills, as well as teaching skills of handling emotional stressors experienced by both the staff and service recipients.

## Introduction

In recent years, workplace violence has become an issue of growing concern in many countries [1]. Of all occupational sectors, healthcare workers, especially nurses, are particularly at risk [2, 3].

Violence experienced by hospital emergency department (ED) personnel has been extensively studied. The staff of these departments are among the most vulnerable to violence from patients and their relatives. EDs are the gateways to all other departments, and are characterized by high workload 24 h a day and high patient turnover. Patients and their relatives are often in a state of severe mental distress and frustration due to the patients’ urgent medical problem, pain, fear of the unknown, and long waits. This in turn may impair their judgment, increasing the likelihood of violence. Indeed, the high incidence of violence in EDs is well-documented [4]. Two previous studies conducted in Israel analyzed the exposure of hospital ED personnel to violence [5, 6]. These studies identified a variety of individual, situational, and interactional factors related to this phenomenon. A major finding pointed at the importance of staff-patient/relative interaction in leading to or being an antecedent of violent incidents [6].

Violence against ED personnel by patients (Ps) and/or their accompanying persons (APs) should be seen as the extreme manifestation of frustration and anger in their interaction with ED personnel. Based on previous research, our assumption is that service recipients’ dissatisfaction with ED services arouses feelings of anger and frustration, which in extreme cases may lead to violence towards ED personnel [7]. In the present study, we shift the focus of attention away from the experiences of actual or potential victims (i.e. ED personnel) to those of actual or potential perpetrators, i.e. Ps and their APs, to better understand the factors related to violent incidents in EDs.

Although quite a number of studies have documented attitudes and feelings of Ps in EDs, more methodologically sound research on this topic is required. Moreover, most studies on this topic have totally ignored APs in their analyses. Thus, it is the aim of this study to conduct in-depth analysis of factors related to both Ps and APs’ negative feelings in EDs.

## Background

Quite a number of recent studies have addressed patients’ (dis)satisfaction with the treatment they received in the ED. This seems to be a universal topic, preoccupying scholars and practitioners alike in various countries. A number of literature reviews have reached very similar conclusions. A review of 20 years of patient satisfaction research identified five major elements of the ED experience that correlate with patient satisfaction: timeliness of care, empathy, technical competence, information dispensation, and pain management [8]. Another review of 50 empirical studies concluded that the most robust predictor of global satisfaction was the quality of interpersonal interactions with the ED provider [9]. A report issued by the Ontario Hospital Association indicated that professional/expert perceptions about what constitutes good quality ED care are not always consistent with patient perceptions; this report emphasized that increases in wait times heighten patients’ anxiety and affect their self-control [10]. A review of 12 qualitative studies reported that the most critical patient experience issue was caring or lack of caring about patients’ psychological and emotional needs, as opposed to the organizational culture of the ED, which emphasized “medical-technical” skill and efficiency [11]. Finally, interpersonal skills/staff attitudes, provision of information or explanations, and perceived waiting times were the most frequently identified service factors in yet another study [12].

A major shortcoming of almost all relevant studies is that they limited their focus to the feelings and attitudes of ED Ps. An important group served by EDs – the APs – is totally absent in these studies. Thus, our focus of attention in the present study is the feelings and attitudes of both Ps and their APs. Based on previous studies, our major hypothesis is that negative feelings (frustration and anger) among ED service recipients are significantly related to the following variables: 1. Staff general attitudes and the quality of the ED experience; 2. Staff specific attitudes towards Ps and APs; 3. Waiting time; 4. Perceived quality of medical care; 5. Information provided to Ps and APs; and 6. Severity of medical problem.

## Method

The present study was part of a larger project on violence against ED personnel in 25 general hospitals in Israel (see also 6, 7) [7]. It examined EDs in seven major hospitals located in different parts of the country, thus increasing the probability that they comprise a representative sample of Israeli general hospitals.

Data was collected by structured face-to-face interviews with Ps and APs. In order to attain a proper representation of the two groups, interviews were timed to cover all days in the week, all shifts of the day, as well as all ED sub-departments. Due to the heterogenic ethnic composition of Israel’s population, interviewers were fluent not only in Hebrew but also in other languages, mainly Arabic and Russian. The interview was designed to assess respondents’ expectations, feelings about the medical and general treatment they received, as well as perceptions regarding violent incidents they witnessed or experienced personally. More specifically, they were asked about waiting for treatment, feelings of stress, helplessness and frustration, and staff attitudes (empathy vs. indifference). The interviews were conducted at the last stage of the medical treatment, i.e., while waiting for the final test results and/or medical staff’s decision – discharge or hospitalization.

### Participants

A total of 354 Ps were personally approached by the interviewers, of which 322 agreed to be interviewed (a 91% response rate). Of the 398 APs approached, 370 participated (93%). The latter were the Ps’ parent (33.9%), son/daughter (25.9%), spouse (16.5%), other family member (11.6%), friend or acquaintance (12.1%). Participants’ basic sociodemographic characteristics are specified in Table 1.

**Table 1:** Socio-Demographic Background Variables of Patients (Ps) and Accompanying Persons (APs)

Variable		Ps		APs		Total		Statistics
		N	%	N	%	N	%	
Gender	Male	173	53.7	114	30.8	287	41.5	χ^2^_(1)_ = 37.25*** (Φ = 0.23)
	Female	149	46.3	256	69.2	405	58.5	
Marital Status	Single	91	28.3	48	13	139	20.1	χ^2^_(3)_ = 46.60*** (r_c_ = 0.26)
	Married	181	56.4	289	78.1	470	68.1	
	Divorced	26	8.1	27	7.3	53	7.7	
	Widow/er	23	7.2	5	1.4	28	4.1	
Age	20 & less	50	15.6	22	6	72	10.5	χ^2^_(3)_ = 27.21*** (r_c_ = 0.20)
	21–50	183	57.2	274	74.5	457	66.4	
	51 & above	87	27.2	72	19.6	159	23.1	
Education	Elementary	58	18	31	8.4	89	13.1	χ^2^_(3)_ = 24.34*** (r_c_ = 0.19)
	Secondary	166	51.6	178	48.1	344	50.7	
	Postsecondary	39	12.1	54	14.6	93	13.7	
	Academic	51	15.8	102	27.6	153	22.5	
Country of Origin	Israel	226	70.2	248	67	474	68.5	χ^2^_(2)_ = 3.25
	Eur./America	48	14.9	74	20	122	17.6	
	Asia/Africa	48	14.9	48	13	96	13.9	
Nationality	Jewish	269	84.6	294	81.2	563	82.8	χ^2^_(1)_ = 1.35
	Non Jewish	49	15.4	68	18.8	117	17.2	
Religiosity	Secular	161	50	188	51.4	349	50.7	χ^2^_(2)_ = 5.26
	Traditional	123	38.2	116	31.7	239	34.7	
	Religious	38	11.8	62	16.9	100	14.5	
Income - compared to average	Much lower	54	31.8	63	29	117	30.2	χ^2^_(4)_ = 3.56
	Lower	22	12.9	41	18.9	63	16.3	
	Same as av.	24	14.1	36	16.6	60	15.5	
	Higher	35	20.6	37	17.1	72	18.6	
	Much higher	35	20.6	40	18.4	75	19.4	

^***^p < 0.001

As can be seen Table 1, some significant differences were found between the two groups:

Gender: Whereas more than half of Ps were males, more than two thirds of APs were females.

Age: Ps were more represented at the lower and higher age groups (up to 20 and 51 and above, respectively), whereas APs were much more represented at the middle age group (21–50).

Marital status: A greater proportion of APs were married, whereas Ps were more represented in the “single” category.

Education: APs had a significantly higher level of education than Ps.

Country of origin: More than two thirds of the participants (69%) were native Israelis, the rest having immigrated from Europe/America (18%) or Asia/Africa (14%).

Nationality: More than eight out of ten participants were Jewish. Among non-Jews, most were Arabs (Muslims and Christians).

Religiosity: About half the respondents defined themselves as secular. Among the rest, Ps tended to be slightly more “traditional” whereas APs were more represented in the “religious” or orthodox category.

Income: Almost half of the respondents (47%) claimed to have a lower or much lower income than the national average and 38% reported their income to be higher or much higher than the average. No differences between the two groups were found on this variable.

Regression analyses revealed that none of the sociodemographic background variables significantly predicted feelings of anger and frustration (negative feelings), whether for the total sample [F_(9,367)_ = 0.82, n.s.], or for Ps [F_(9,156)_ = 0.89, n.s.] and APs [F_(9,201)_ = 1.40, n.s.].

### Measures

During the face-to-face interviews, interviewers filled structured questionnaires of Ps and APs. The questionnaires of the two groups were almost identical with only few differences, due to the different perspective of each of group.

The questionnaires each contained 40 items, of which 20 had a yes/no scale, 14 had a 1 (bad) to 5 (very good) scale, two had a 1 (less than 15 min) to 5 (more than 3 h) scale, one had a 1 (not noisy at all) to 5 (very noisy) scale, one had a 1 (up to half an hour) to 6 (more than 6 h) scale, one had a 1 (moderate medical problem) to 5 (life-threatening medical problem) scale, and one had a 1 (no pain) to 6 (very serious pain) scale. Their respective Cronbach alphas were 0.81 (Ps) and 0.83 (APs).

The questions asked were grouped into eight major (aggregate) independent variables, related to the respondents’ experience in the ED:

1. *General attitudes of staff and quality of ED experience* included quality of ED service, quality of the department, noise in ED, registration efficiency, receptionist attitudes, privacy, waiting space for APs, and cleanliness.
2. *Staff attitudes towards patient:* nurses and doctors’ general attitudes, whether they understood the P (for both P and AP), patience with the P (for both P and AP), respect for the P (for both P and AP), disturbance of the P (only for P) and patronizing the P (only for AP).
3. *Staff attitude towards the AP*: staff disturbance of the AP, understanding the AP, patience with the AP, respect for the AP and/or patronized the AP (all only for AP).
4. *Waiting* for nurse, doctor, promptness, and total time in ED.
5. *Perceived quality of medical care* by nurses, doctors, and their professionalism (only for AP).
6. *Information provided to P & AP* that calmed or explained the situation, explained the treatment (all for both P and AP), and whether the AP was a partner in the decision on treatment (only for AP).
7. *Information provided to patient* (as reported by AP) and whether the P was a partner in the decision on treatment.
8. *Severity of medical problem* and severity of pain.

The above eight aggregate variables were the independent variables. The dependent variable was “reported negative feelings experienced while at the ED” (hereafter, “negative feelings”). The construction of the negative feelings scale and the range of its scores are specified in Appendix. There was no significant difference between Ps’ and APs’ scores on that variable [t(690) = −0.77, n.s.]. Data were analyzed separately for the two groups of respondents.

## Results

Negative feelings while in the ED were reported by 23.6% of patients (76 out of 322) and by 20.5% of APs (76 out of 370). Our basic assumption is that within this group of frustrated and angry clients lies the potential for violent outbursts against ED staff. Since witnessing violent incidents can amplify feelings of stress and anxiety, it is no coincidence that half (50%) of the Ps with negative feelings and 40% of the APs with negative feelings reported of having witnessed violence while in ED, compared to only 22% and 19% (respectively) of respondents without negative feelings. Moreover, among those with negative feelings, 12% of Ps and 5% of APs admitted that they themselves had behaved violently in the ED (shouting at, cursing, or threatening a staff member), in comparison to only less than 1% of respondents without negative feelings. Reports of being victimized were found only among respondents with negative feelings: 4% among Ps and 5% among APs.

Therefore, it is of both theoretical and practical importance to analyze the factors related to negative feelings among the Ps and APs in the EDs. A preliminary regression analysis (conducted separately for Ps and APs) revealed that almost all individual items were significantly related to the dependent variable, as predicted.

### Statistical analyses

The data were analyzed using the Predictive Analytics Software (PASW, Version 21.0). Simple and multiple regression analyses, with Bonferroni correction, were used to examine the effects of the independent variables on the dependent variable – negative feelings of Ps and APs. Our aim was to find out which of the above aggregate variables were the best predictors of Ps and APs’ negative feelings.

Significance was set at the .05 level and all tests of significance were one-tailed.

#### Patients (Ps)

The six aggregate variables that related to patients only were constructed by averaging all the items that related to each. The regression model was found to be significant [F_(6,315)_ = 19.43, *p* < 0.001], explaining 25.6% of the variance of negative feelings. None of the independent variables showed evidence of multicollinearity (Tolerance >0.32). The findings indicate that of all independent variables, the only significant predictor of patients’ negative feelings was *perceived quality of medical care* (B = −9.14; β = −0.35; *t* = 4.21, *p* < 0.001).

At the second stage, we examined which of the variables comprising perceived quality of medical care predicted negative feelings. Also, although when analyzed together the other five major variables were insignificant predictors of negative feelings, it is of theoretical and practical interest to examine which of the individual items in these five major variables were significantly related to negative feelings, when analyzed separately. All these results are presented in Table 2.

**Table 2:** Summary of significant results related to patients

Variable	Predictor	B	β	t	F
1. Staff’s general attitudes and quality of ED experience	1. Low quality of service	−3.96	−0.23	2.30^*^	F_(8,228)_ = 8.80^***^ R^2^ = .209 Tolerance >0.33
	2. Low quality of department	−4.39	−0.23	2.33^*^	
2. Staff attitudes towards patient	1. Nurses’ negative attitude	−5.81	−0.30	5.00^***^	F_(6,296)_ = 28.65^***^ R^2^ = .355 Tolerance >0.35
	2. Staff’s Impatience	−11.81	−0.21	2.77^**^	
	3. Staff’s disturbance	6.37	0.16	3.28^**^	
4. Waiting	Lack of promptness	−4.47	−0.10	3.28^***^	F_(4,313)_ = 9.96^***^ R^2^ = .102 Tolerance >0.86
5. Perceived quality of medical care (xx)	1. Low perceived quality of medical care by nurses	−0.78	−0.34	5.45^***^	F_(2,298)_ = 54.25^***^ R^2^ = .262 Tolerance >0.64
	2. Low perceived quality of medical care by doctors	−0.59	−0.24	3.94^***^	
6. Providing information to patient	Not explaining the situation	−6.68	−0.44	3.28^**^	F_(5,76)_ = 3.25^*^ R^2^ = .122 Tolerance >0.57
8. Severity of medical problem	Low severity of patient’s medical problem	−2.67	−0.14	2.36^*^	F_(2,306)_ = 5.34^**^ R^2^ = .027 Tolerance >0.88

^***^ *p* < 0.001, ^**^ *p* < 0.01, ^*^ *p* < 0.05, (xx) Significant predictor of negative feelings

The results in Table 2 indicate that patients’ negative feelings were significantly related to the following variables:

1. Low quality of service and department (Var. 1);
2. Nurses’ negative attitudes, staff’s impatience and their disturbance (Var. 2);
3. Lack of promptness (Var. 4);
4. Low perceived quality of medical care by nurses and doctors (Var. 5);
5. Insufficient explaining of the situation (Var. 6); and
6. Low severity of Ps’ medical problem (Var. 8).

#### Accompanying persons (APs)

Here too, we first examined which of the major (aggregate) eight variables significantly predicted negative feelings among APs. The regression model was found significant [F_(8,263)_ = 16.08, *p* < 0.001], explaining 30.8% of the variance of negative feelings. None of the independent variables showed evidence of multicollinearity (Tolerance >0.51).

Three variables were found to significantly predict APs’ negative feelings: *(1) Staff’s general attitudes and quality of treatment* (B = −10.79; β = −0.28; *t* = 4.61, *p* < 0.001), i.e., negative attitudes and low quality of treatment were related to APs’ negative feelings; *(2) Staff’s attitude towards patient (*B = −24.04; β = −0.38; *t* = 5.63, *p* < 0.001), here too, negative attitude towards patient predicted AP’s negative feelings; and (3) *Severity of patient’s medical problem* (B = −1.92; β = −0.12; *t* = 2.29, *p* < 0.05), i.e., the less severe the medical problem, the higher the APs’ dissatisfaction.

At the second stage, we examined which of the items included in the three above- mentioned variables significantly predicted negative feelings among APs. Here too, we also examined within each of the five insignificant aggregate predictors, which of their respective items are significantly related to negative feelings, when analyzed separately. These results are presented in Table 3.

**Table 3:** Summary of significant results relating to accompanying persons (APs)

Variable	Predictor	B	β	t	F
1. Staff’s general attitude and quality of ED experience (xx)	1. Low quality of service	−6.56	−0.43	6.31^***^	F_(8,300)_ = 18.94^***^ R^2^ = .318 Tolerance >0.45
	2. Lack of privacy	−4.57	−0.22	4.23^***^	
	3. Waiting space for AP	1.85	0.13	2.44^*^	
2. Staff attitudes towards P (as perceived by AP) (xx)	1. Doctors’ negative attitude	−7.69	−0.32	5.93^***^	F_(6,329)_ = 30.14^***^ R^2^ = .343 Tolerance >0.46
	2. P not understood by staff	−30.24	−0.38	6.21^***^	
3. Staff attitude towards AP	1. AP disturbed by staff	12.16	0.29	6.14^***^	F_(5,340)_ = 37.98^***^ R^2^ = .349 Tolerance >0.55
	2. AP not understood by staff	−21.21	−0.28	4.75^***^	
	3. AP patronized by staff	12.40	0.17	3.53^***^	
4. Waiting	1. Waiting for doctor	1.94	0.15	2.83^**^	F_(4,353)_ = 29.78^***^ R^2^ = .244 Tolerance >0.74
	2. Lack of promptness	−6.61	−0.42	7.94^***^	
5. Perceived quality of medical care	1. Low quality by nurse	−2.25	−0.13	2.45^***^	F_(3,316)_ = 63.33^***^ R^2^ = .370 Tolerance >0.65
	2. Low quality by doctor	−4.80	−0.20	3.73^***^	
	3. Low degree of professionalism	−33.21	−0.44	9.06^***^	
6. Providing information to AP	Not explaining the situation	−6.75	−0.41	3.85^***^	F_(5,137)_ = 3.79^**^ R^2^ = .089 Tolerance >0.55
7. Providing information to P (reported by AP)	Lack of information to P	−11.36	−0.26	3.05^**^	F_(2,240)_ = 15.24^***^ R^2^ = .105 Tolerance >0.50
8. Severity of medical problem (xx)	Low severity of patient’s pain	−1.23	−0.12	2.18^*^	F_(2,359)_ = 4.54^*^ R^2^ = .019 Tolerance >0.91

^***^ *p* < 0.001, ^**^ *p* < 0.01, ^*^ *p* < 0.05, (xx) Significant predictor of negative feelings

The results in Table 3 indicate that APs’ negative feelings were significantly related to the following variables:

1. Low quality of service, lack of privacy, and availability of waiting space^1^ for APs (Var. 1);
2. Doctors’ negative attitudes, and P not being understood by staff (Var. 2);
3. Staff disturbing AP, not understanding, and patronizing him/her (Var. 3);
4. Long wait for doctors and lack of promptness (Var. 4);
5. Low perceived quality of medical care by nurses and doctors and low degree of professionalism (Var. 5);
6. Not explaining the situation to AP (Var. 6);
7. Lack of information to P (Var. 7); and
8. Low severity of P’s pain (Var. 8).

### Summary of significant results

The results provide only partial support for our major hypothesis: the multivariate analysis of all aggregate variables revealed that among Ps, the single significant predictor of negative feelings was low perceived quality of medical care. Among APs, three predictors of negative feelings were identified: staff’s general attitudes and quality of ED experience, attitudes towards P, and low severity of P’s medical problem. The analyses of items of the individual variables revealed both similarities and differences between Ps and APs, which are discussed below. Note that with the exception of a single item (waiting space for APs), all significant results were in the predicted direction.

## Discussion

This study analyzed the factors related to negative feelings of patients (Ps) and their accompanying persons (APs) in the emergency departments (Eds) of seven major hospitals in Israel. A major finding is that dissatisfaction with ED services is quite a common phenomenon – it is expressed by about 1 in 4 Ps and 1 in 5 APs. These negative feelings constitute the emotional reservoir that feeds violent outbursts in the form of verbal and/or physical attacks against ED personnel.

Another major contribution of this study to the literature is the inclusion of both Ps and their APs in the analyses. This original design enabled us to take into account the entire population served in EDs, not only Ps, as in most previous studies.

Basically, we found that APs adopt a different and broader perspective than that of patients (see Table 3). Being mostly (76.3%) first-degree relatives (parents, children or spouses), APs consider themselves the Ps’ guardians, protectors and representatives in the stressful and bureaucratic ED environment, given Ps’ often impaired judgment due to pain, suffering, and fear of the unknown.

The different perspectives demonstrated by Ps and APs are mainly evident in the results of the multivariate analysis of the aggregate variables. This analysis reveals that Ps adopt a narrowly focused perspective, singling out low perceived quality of medical care (by both doctors and nurses) as the only significant source of their negative feelings. As the recipients of medical care, they understandably perceive it as being of utmost importance. All other factors seem secondary in comparison.

APs’ perspective, on the other hand, is broader and encompasses a combination of medical and attitudinal factors, including staff’s general attitude and quality of ED experience (e.g., low quality of service, lack of privacy), negative attitudes towards the P (doctors’ attitudes, P not understood), and severity of the P’s pain. These variables point at the APs’ deep empathy to and involvement with the health and wellbeing of their dear ones treated in the ED. This broader perspective expressed by APs is also reflected in the generally higher percentage of explained variance (R^2^) found in their regressions of both the aggregate variables and most of the individual ones (see Tables 2 and 3).

Combining the significant variables of both Ps and APs in EDs, we can conclude that the important elements that concern them and contribute to their negative feelings are:

- Low quality of department and service, including lack of privacy;
- Medical staff’s negative attitudes, as well as their impatience, disturbance, lack of understanding and patronizing attitude;
- Lack of promptness and long waiting;
- Low perceived quality of medical care;
- Failing to explain the situation and providing insufficient information;
- Low severity of P’s medical problem.

With regard to the last item, note that both Ps and APs express less negative feelings in cases of severe medical problems. It seems that in these cases, the medical and other staff make greater effort to attend to the patient’s situation, and as a result, are more appreciated by Ps and APs.

As can be seen quite clearly, most of our findings are in line with those of a number of previous studies mentioned earlier. The relatively high proportion of dissatisfaction among ED service recipients indicates that any policy aiming at violence reduction needs to address dissatisfaction with ED services as a structural/general issue, beyond the individual (actual or potential) perpetrators.

Studies concerning the interpersonal discourse in medical encounters suggest the possibility of maintaining effective and affective interpersonal communication, which can ventilate frustration and anxiety [13]. The evolving concept of “patient-centeredness” is becoming more and more operationalized and feasible as a practical framework for the restoration of patients’ sense of agency and empowerment, and for the construction of an effective interpersonal and citizen-state discourse [14, 15].

A second implication of our findings has to do with APs. Most laws concerning patients’ rights do not include the rights of patients’ families or other APs, though they are major actors in the interaction between the patient and the health service providers. In practice, the APs take a major part in the decision-making process. Specific legislation or regulations are needed to stipulate the APs’ rights, especially when the patient is in great distress and needs a supportive figure nearby.

A final recommendation has to do with interpersonal training. In many medical training schools, there are no (or not enough) courses on interpersonal and human service skills aiming at developing awareness of the emotional state of service recipients, as well as teaching skills of handling emotional stressors experienced by both the staff and service recipients, and skills of reflection on action and reflection in action [16]. In order to improve and maintain the staff’s reflective and interpersonal skills, we recommend an on-job training mechanism within hospitals, under the Ministry of Health’s responsibility. Following the Israeli law on the prevention of violence in care institutions [17], we recommend implementing the guidelines of the Director General of the Ministry of Health [18] related to the professional training of staff such as doctors and nurses, to cope with stressful interpersonal situations and acquire skills of stress reduction and de-escalation of conflicts with service recipients.

Finally, we would like to point at the major limitation of this study: its limited scope, in terms of both the number of hospitals and the number of respondents included. More research is needed, in line of our design and methodology, both in Israel and in other countries, to assess the generalizability of our findings.

## Conclusions

Besides broadening the scope of attention to a hitherto neglected type of ED service recipients (APs), our research identified the important elements affecting ED service recipients’ negative experiences. These results have several practical implications as presented in the Discussion, and may serve as guidelines for improving ED service recipient-staff communication. A possible path for achieving this goal is by taking advantage of the cooperation of APs in improving Ps’ general experience while being served in EDs. Reducing Ps and APs’ negative feelings will in turn contribute to the reduction of their violent outbursts against ED personnel.

## Appendix

### Construction of the Negative Feelings Scale

This scale was based on the participants’ responses to nine yes/no items relating to their feelings during their visit to the ED. The instructions to participants were as follows:

*The following table describes various feelings service recipients experience while in the ED. Please indicate which of these feelings you experienced.** ***.***

**Table:**

	Yes (1)	No (0)
1. Relaxed regarding the treatment I (P)** received		
2. In control regarding the treatment I (P) received		
3. Worried regarding the treatment I (P) received		
4. Helpless regarding the treatment I (P) received		
5. Stressed regarding the treatment I (P) received		
6. Frustration/anger regarding the attitude towards me (P)		
7. Frustration/anger regarding the treatment I (P) received		
8. An urge to burst out at someone of the staff because of his/her attitude towards me (P)		
9. An urge to burst out at someone of the staff because of the treatment I received***		

*The interviewers made it clear that these items do not relate to the feelings (pain, worries, etc.) that caused the admission to the ED, but to the quality of treatment & attitude received by ED staff,

**In questionnaires for APs.

***For APs question 9 was: An urge to burst out at someone of the staff because of his/her attitude towards me.

The total individual score was calculated by counting the Yes (1) answers to the negative feelings (items 3–9), and subtracting the Yes (1) answers to the positive feelings (items 1–2). The final scores ranged from −2 (only positive feelings, no negative ones) to 7 (only negative feelings, no positive ones).

## Abbreviations

AP: Accompanying person
ED: Emergency department
P: Patient

## Footnotes

1 The result regarding this item contradicts our hypothesis
